# Selective and non-selective carotid ultrasound screening and perioperative stroke incidence in the coronary artery bypass grafting population: a systematic review and meta-analysis

**DOI:** 10.1186/s13019-026-04616-6

**Published:** 2026-07-19

**Authors:** Ben King-O’Reilly, Daniel Lewis Graves, Kabeshan Sandrasekaran, Norman Briffa

**Affiliations:** 1 Cardiothoracic Department, The University of Sheffield Medical School, Sheffield, England; 2 Sheffield Teaching Hospitals NHS Foundation Trust Cardiothoracic Centre: Northern General Hospital Cardiothoracic Centre, Sheffield, England; 3 Sheffield Medical School, University of Sheffield, Beech Hill Road, Sheffield, S10 2RX England

**Keywords:** Carotid Stenosis, Coronary artery bypass grafting, Carotid ultrasound, Screening, Perioperative stroke, Meta-analysis

## Abstract

**Objectives:**

A systematic review and meta-analysis was performed to observe the incidence of perioperative stroke associated with selective and non-selective carotid ultrasound (CU) screening in the isolated coronary artery bypass grafting (CABG) population.

**Methods:**

PubMed, Scopus and CENTRAL databases were searched for studies published between January 1995 and October 2025. Inclusion criteria for the review focused on studies reporting primary data related to perioperative stroke in adult patients scheduled to undergo non-emergency isolated CABG who underwent either selective or non-selective preoperative CU screening. The primary outcome was perioperative stroke. Study quality was assessed using the ROBINS-I tool. Data related to the primary outcome was synthesised using an exploratory random-effects model meta-analysis. This review was reported as per PRISMA guidelines.

**Results:**

2398 studies were identified to undergo title and abstract screening; 63 studies were identified to undergo full-text screening of which 15 studies were eligible for inclusion within the review (Total N = 25,931). For the 13 studies utilising a non-selective screening strategy, the pooled perioperative stroke rate was 1.78% (95% CI: [1.28;2.48]). Independently, the two cohorts utilising a selective screening strategy reported perioperative stroke rates of 1.16% and 1.23%. Due to the structural limitations of the existing literature these findings are presented as independent descriptive cohorts.

**Conclusions:**

This review provides a descriptive synthesis of perioperative stroke rates across the two previously mentioned screening cohorts and does not establish comparative effectiveness. Although the low absolute event rates observed within the selective screening cohort align with current guideline recommendations, the structural limitations and high disproportion of the available evidence base prevent definitive comparison.

**Supplementary Information:**

The online version contains supplementary material available at https://doi.org/10.1186/s13019-026-04616-6.

## Introduction

Coexisting extracranial carotid artery disease (ECAD) and coronary heart disease (CHD) commonly affects patients with cardiovascular diseases as these diseases both share similar atherosclerotic mechanisms [1, 2]. As a result, around 8% of patients with CHD severe enough to warrant coronary artery bypass grafting (CABG) have severe ECAD. Presence of symptomatic or asymptomatic ECAD is an independent predictive factor for both major adverse cardiac events and neurological events, such as cerebrovascular accidents (CVAs) and transient ischaemic attacks (TIAs) [1, 3]. Symptomatic ECAD is defined as an acute neurologic deficit, including CVAs or TIAs, referable to ipsilateral carotid atherosclerotic pathology which has occurred in the preceding six months [4, 5]. Asymptomatic ECAD is defined as a ≥ 50% narrowing of the carotid artery in the absence of cerebral ischemia in the preceding six months [6].

Treating coexisting ECAD and CHD has historically been problematic with there being a 1.4% to 3.8% risk of CVA in patients undergoing CABG to treat CHD and a 0.0–2.0% risk of myocardial infarction (MI) in patients undergoing carotid endarterectomy (CEA) to treat ECAD [7]. As a result, many attempts have been made to identify the optimal method for treating coexisting ECAD and CHD which minimises the risk of MI and neurological complications [7]. Current treatment methods include simultaneous CABG and CEA procedures; staged procedures (either CABG with subsequent CEA or vice versa); and transcatheter procedures incorporating percutaneous coronary intervention (PCI) or carotid artery stenting (CAS) [7]. However, the optimal management strategy for coexisting ECAD and CHD remains a subject of debate with guidelines such as those of the American Heart Association (AHA) stating the strategy for carotid and coronary revascularisation should be dictated by the absence or presence of given clinical symptoms [7, 8].

Perioperative stroke, also known as perioperative CVA, remains a devastating complication following CABG, with the incidence of perioperative CVA in the general CABG population being in the range of 1.4% to 3.8% [7]. While embolic events often arise from surgical manipulation and clamping of the ascending aorta, ECAD represents a distinct and independent risk factor [9]. In patients with significant ECAD, CVA may occur due to intraoperative hypoperfusion during cardiopulmonary bypass or via a thromboembolism originating directly from the carotid plaque [10]. As a result, the risk of perioperative CVA in the CABG population presents surgeons with a dilemma when treating patients with coexisting ECAD and CHD, especially in patients with significant asymptomatic ECAD.

Carotid ultrasound (CU) is a repeatable and non-invasive procedure which preoperatively assesses CVA risk related to ECAD by identifying and quantifying the size and characteristics of plaques within the carotid arteries, this facilitates informed decisions regarding the necessity of prophylactic carotid interventions, such as CEA or CAS [11–15]. However, universal, non-selective preoperative CU screening of the CABG population, specifically patients with asymptomatic ECAD, remains controversial with current guidelines not supporting the practice [11–14]. This hesitancy is partially due to a lack of clear evidence supporting non-selective CU screening, as well as the 92% specificity of CU scanning which can lead to unnecessary procedural risks via false positive screening results [8, 13, 14, 16].

Although non-selective CU screening of the CABG population is not recommended, selective CU screening of high-risk CABG patients, such as in patients with a prior history of CVA or TIA, is common practice [8, 11–14]. However, the application of screening in patients undergoing CABG with asymptomatic ECAD remains inconsistent [11–14]. This discrepancy is particularly noteworthy as these patients exhibit levels of ECAD significantly higher than that of the general population and are scheduled for a procedure which independently compounds their risk of perioperative CVA [9, 10, 17, 18].

The primary reason guidelines do not generally recommend screening CABG patients with asymptomatic ECAD is the current lack of clear high-quality randomised controlled trials (RCTs), directly comparing outcomes between non-selective and selective screening strategies [8, 13, 14].

This systematic review aims to synthesise the best available evidence to determine the effectiveness of non-selective and selective screening strategies and their subsequent management strategies on all-cause mortality and perioperative CVA incidence.

Equally, this review also aims to determine the rates of major complications resulting from prophylactic interventions triggered by positive screening results.

Although the management of patients with symptomatic ECAD is dictated by established clinical trials, this review aims to fill a critical evidence gap by exploring the extent to which the two prior mentioned preoperative screening strategies can be used to successfully inform clinical decision-making and ultimately improve the management of patients with asymptomatic ECAD and as a result the perioperative outcomes of the wider isolated CABG population.

## Methods

This systematic review was registered with PROSPERO, CRD420251169400. Following protocol amendments to refine the initially restrictive eligibility criteria, initially collected data was discarded prior to recommencing and reporting the review as per PRISMA guidelines [19]. Ethical approval was not required for this review as it was based on published literature.

### Study group definitions

Studies were classified as using a selective and / or non-selective screening strategy based on the following definitions:

- Selective screening: the practice of restricting the use of preoperative CU scanning to a clinical subset of patients demonstrating high-risk indicators, defined as: symptomatic ECAD, including prior TIA and CVA; the presence of a carotid bruit or non-palpable carotid pulse; a history of peripheral vascular disease; severe left main disease; or advanced age (greater than 60 years). Patients lacking these specific risk factors were not screened.
- Non-selective screening: the practice of performing preoperative CU screening on all patients scheduled to undergo isolated CABG regardless of the presence or absence of given risk factors.

### Search strategy

A literature search was performed across the PubMed, Scopus and CENTRAL databases, identifying studies related to our PICO elements which were published between 1st January 1995 and 27th October 2025. The search strings utilised for each database, presented in Supplementary Material, Table S1, were derived from our PICO with the aim of maximising sensitivity to ensure all potential evidence was captured across multiple databases.

### Eligibility criteria

Eligible studies met the inclusion criteria without violating any of the exclusion criteria as per Table 1. If the study being assessed was a single-arm study, only one of the groups of “Intervention” or “Comparison” criteria were assessed, depending on whether the study utilised a selective or non-selective screening strategy. It should be noted that studies with mixed CABG populations such as those including patients undergoing emergency CABG or CABG combined with other non-carotid related procedures were eligible for inclusion provided sufficient sub-group analysis providing uncontaminated data of the non-emergency isolated CABG population was available.

**Table 1:** Summary table of the inclusion and exclusion criteria utilised in the study selection process

PICO	Inclusion criteria	Exclusion criteria
Population	• Human adult male and female patients (over 18 years of age) scheduled to undergo isolated elective or urgent CABG	• Patients scheduled to undergo CABG combined with other major procedures and / or emergency CABG • Studies focusing on patients undergoing non-CABG procedures • Non-human patients • Adolescent patients (under 18 years of age)
Intervention	• Studies must use carotid ultrasound / duplex /doppler as the primary assessment tool • Studies must screen the entire population screen / almost all of the population (provided the ratio of the population screened is provided) • Studies must report ultrasound results of stenosis severity using validated and standardised criteria (e.g. NASCET method, ECST method or local lab criteria) • Studies must include a pre-defined clinical pathway linking a positive ultrasound result to a secondary intervention, such as carotid endarterectomy, stenting, a modified surgical technique, e.g. staged or synchronous surgical approaches, or referral to a specialist to determine the appropriate course of action	• Studies using non-carotid ultrasound techniques as the primary assessment tool, e.g. carotid angiography • Studies where carotid ultrasound is performed during intraoperative monitoring or post-operatively as opposed to as a preoperative assessment tool • Studies where all patients receive carotid ultrasound and routine, prophylactic secondary intervention, such as carotid endarterectomy, regardless of clinical findings
Comparison	• Studies where only patients with a prior history of CVA or TIA receive systematic preoperative carotid ultrasound screening, allowing studies where carotid ultrasound was performed on a small, clinically indicated subset of patients with no prior history of CVA or TIA, such as patients with a physical carotid bruit on exam, however, this must be documented as standard practice	• Studies where patients with no prior history of CVA or TIA receive routine preoperative screening with any carotid imaging modality, not just carotid ultrasound, allowing studies where carotid ultrasound was performed on a small, clinically indicated subset of patients with no prior history of CVA or TIA, such as patients with a physical carotid bruit on exam, however, this must be documented as standard practice • Studies where all patients receive carotid ultrasound and routine, prophylactic secondary intervention, such as carotid endarterectomy, regardless of clinical findings
Study design	• Randomised controlled trials (RCTs), comparative non-randomised studies, such as cohort and case–control studies which directly compare the incidence of perioperative stroke between the two defined prior discussed management strategies and single arm studies which provide results about the outcomes of using one of the two prior discussed management strategies	• Diagnostic accuracy studies aiming to test the accuracy of carotid ultrasound against a "gold standard" test • Studies with insufficient data • Studies from grey literature sources, such as clinical trial registries and thesis and dissertation repositories • Abstract only / Full text is unavailable • Systematic Reviews and Meta-Analyses • Narrative Reviews • Qualitative studies • Case reports • Books • Conference abstracts / proceedings
Other criteria	• Studies must be published from 01/01/1995 • Studies must be published in English Note: A study containing a mixed cardiac surgery and / or emergency and non-emergency population can be included provided there is sufficient sub-group analysis of the desired groups such that there is no contamination of the required data to carry out the review	

### Study selection process

The study selection process utilised a two stage, two-reviewer screening process. Both reviewers (BKOR and DG) independently performed title and abstract screening followed by full-text screening, using the given study, the eligibility criteria in checklist form and a spreadsheet to record outcomes of the screening process for each study. In title and abstract screening, a study would proceed to full-text screening if either reviewer labelled it as either “Include” or “Unsure”. In full-text screening a study would only be deemed eligible for inclusion within the review if both reviewers labelled it as “Include”. Any discrepancies between the inclusion and exclusion decisions of the reviewers were decided by a third reviewer (KS).

### Data extraction process

The data extraction process utilised a two-reviewer extraction process, consisting of both reviewers (BKOR and KS) independently performing data extraction of the included studies by using the given study and a standardised data extraction form. Any discrepancies between data extracted by the two reviewers were decided by a third reviewer (DG). Extracted data included study characteristics, population demographics and specific details of the studies’ CU screening strategy, such as the percentage ECAD threshold for a positive screening result and the pre-defined clinical pathway triggered upon identification of a positive screening result. In this context we define “triggering a pre-defined clinical pathway” to mean upon the discovery of a significant lesion an institutional protocol was initiated resulting in a clinical evaluation taking place to determine the appropriateness of a staged, simultaneous or medical approach.

### Primary and secondary outcomes of the data extraction process

The primary outcome with regards to data to be extracted was obtaining the total number of patients scheduled for non-emergency isolated CABG in each study as well as the total number of perioperative CVA events which occurred in each study. We define perioperative stroke/CVA as a CVA event that lasts for over 24 h, occurring during surgery or within 30 days postoperatively that is confirmed by imaging.

In addition to these primary outcomes, we also sought to extract data regarding the total number of all-cause mortality events which occurred in each study and data regarding the incidence of complications associated with interventions resulting from triggering of the pre-defined clinical pathway associated with a positive screening result, such as CEA or CAS related complications.

### Study quality assessment process

The quality of non-RCT studies was assessed using the risk of bias in non-randomised studies of interventions tool (ROBINS-I) [20]. For each study two reviewers (BKOR and KS) independently assessed risk of bias for the primary outcome of perioperative CVA incidence and secondary outcome of perioperative all-cause mortality incidence. Any discrepancies between the study quality assessment decisions of the reviewers were decided by a third reviewer (DG). Each domain, and the overall risk of bias judgements, were categorised as having “No information” or being at a “Low”, “Moderate”, “Serious”, or “Critical” risk of bias. Results from the study quality assessment process are summarised in Fig. 2 as a traffic-light plot which was generated using the robvis tool [21]. Studies deemed to be at an overall critical risk of bias were excluded from meta-analysis.

### Statistical analysis

All studies meeting the inclusion criteria were eligible for data synthesis for the primary outcome of perioperative CVA rates upon transformation of extracted data from percentages to absolute event counts where applicable.

Statistical analyses were performed using R statistical software (v4.5.2). The meta-analysis was conducted using the metaprop function (meta package). AI-based tools were used to assist with code development, with all analyses, interpretations, and conclusions being verified by the authors.

Given the anticipated clinical and methodological heterogeneity of the included studies a random-effects model was utilised when pooling perioperative CVA rates between studies. Inter-study variance, τ^2^, was estimated using the DerSimonian-Laird method. Individual study confidence intervals (CIs) were calculated using the Clopper-Pearson method. Pooled estimates were derived from logit via logit transformation and reported as percentages with 95% CIs. Statistical heterogeneity was assessed using Cochran’s Q test and the *I* ^2^ statistic. Due to the low number of studies evaluating a selective screening strategy meta-analytic pooling, sensitivity analysis and subsequent publication bias assessments were restricted to the non-selective screening cohort. The sensitivity analysis was performed to identify significant contributors to heterogeneity and to assess the stability of the pooled perioperative CVA rate within the non-selective screening cohort. The publication bias assessment for the pooled non-selective screening cohort was evaluated via funnel plot inspection and Egger’s regression test.

No direct comparative meta-analysis was performed between the non-selective and selective screening cohorts due to the included studies all consisting of independent single-arm non-comparative cohorts which prevent direct hypothesis testing from being statistically reliable.

## Results

### Study identification

**Fig. 1:**
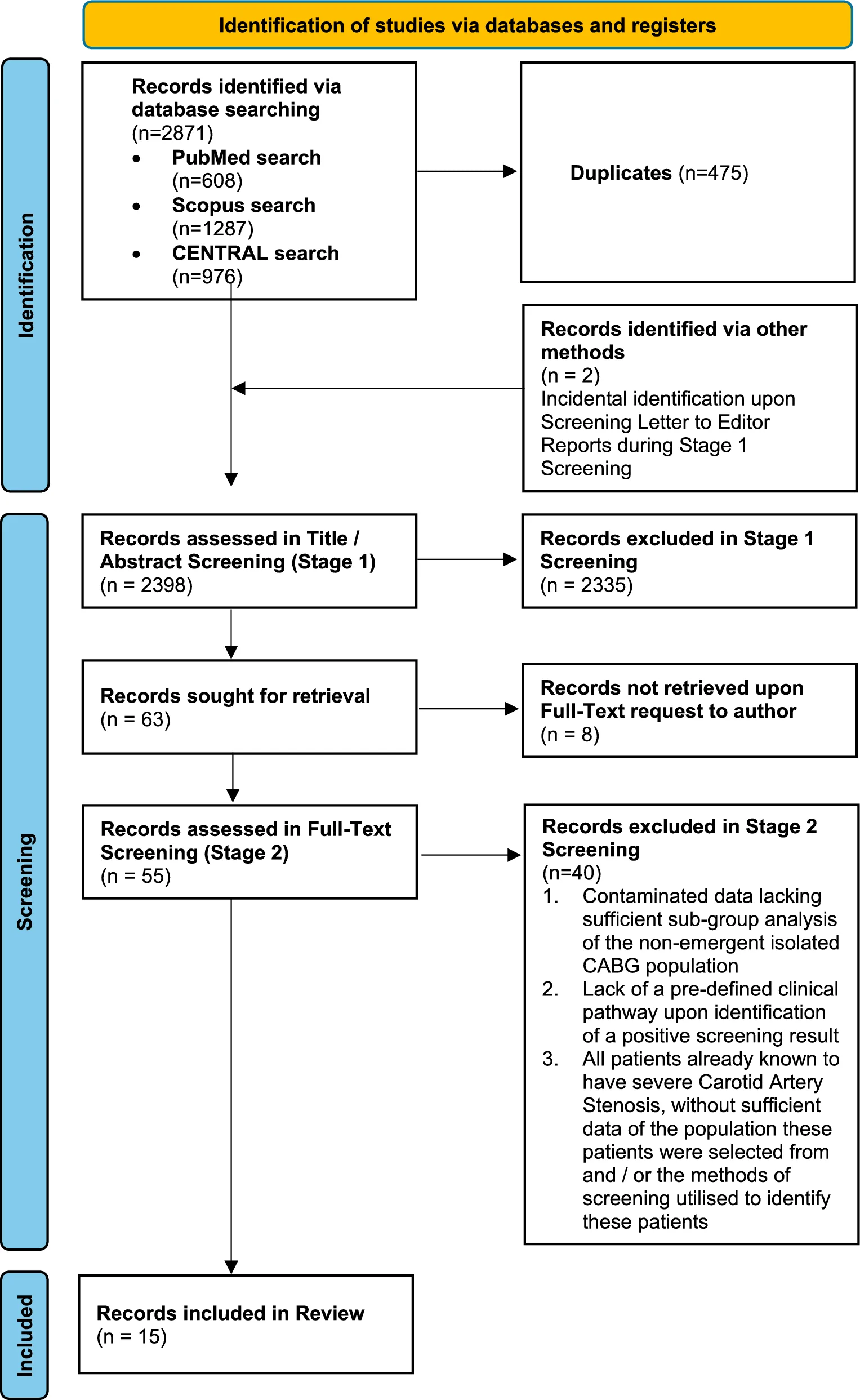
PRISMA flow diagram outlining the outcomes of the study selection process

After deduplication, 2398 records were identified for screening, with 2396 of these records identified via database searches in PubMed, Scopus and CENTRAL, as well as 2 records being incidentally identified during title and abstract screening. After completion of title and abstract screening a remaining 63 records proceeded to full-text screening, with 8 of these full-texts being unavailable to access despite contacting the corresponding authors. Of the accessible 55 full-texts, 15 studies were eligible for inclusion [22–36]. Common reasons for exclusion included contaminated data sets such as those consisting of populations undergoing isolated CABG and CABG combined with valve replacement, lack of a pre-defined clinical pathway linked with positive screening results and lack of information regarding how patients with known severe ECAD were identified and the population from which they were selected. A summary of the study identification process is presented in PRISMA flow diagram format in Fig. 1.

### Study quality assessment result

**Fig. 2:**
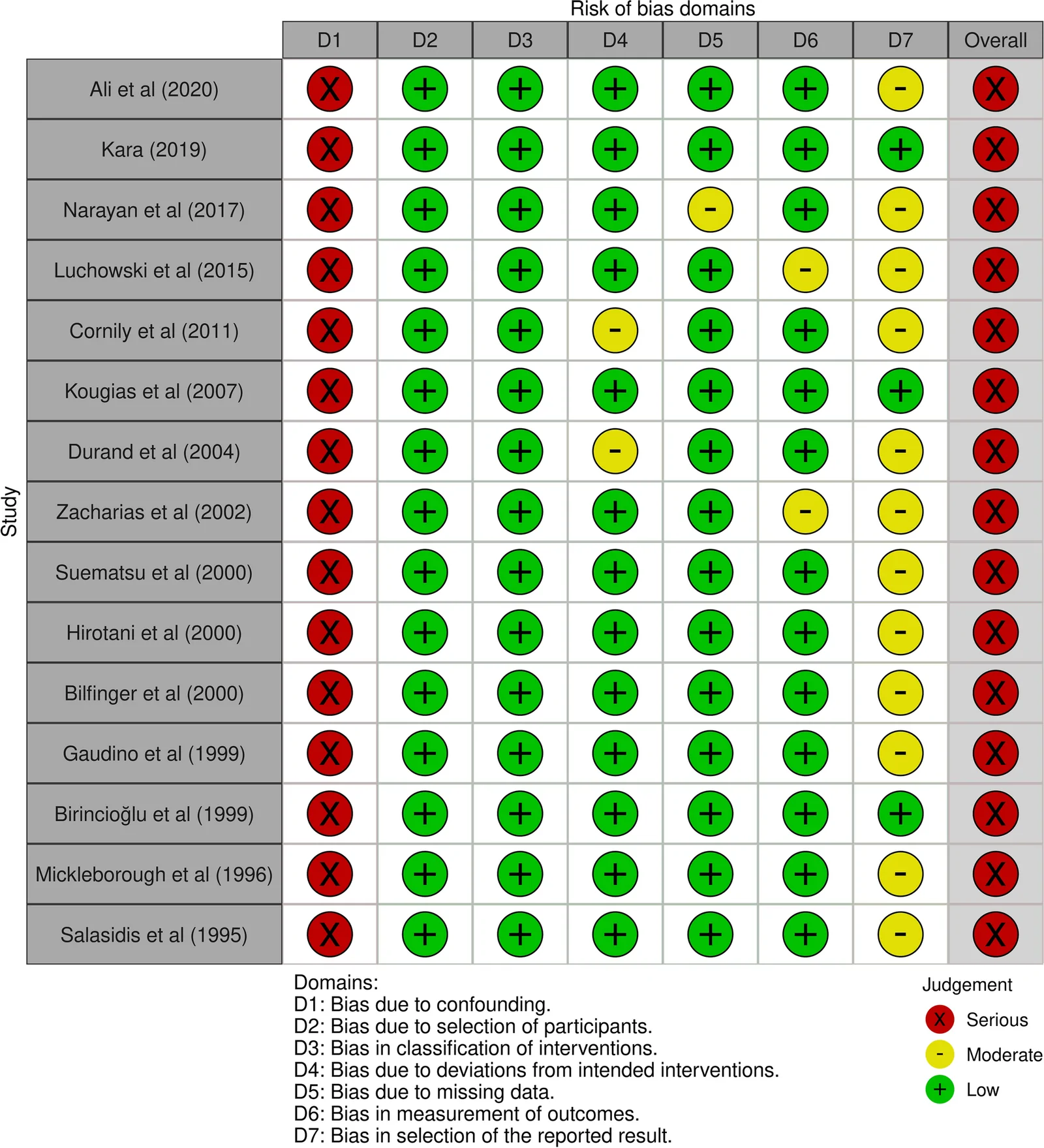
ROBINS-I study quality assessment traffic light plot summary

Results from the study quality assessments are summarised in Fig. 2, detailed justifications for each domain judgement are provided in the Supplementary Material, ROBINS-I Justifications.

All 15 of the included studies were single-arm, non-comparative studies with regards to the preoperative CU screening strategies utilised. As a result, these studies were all uniformly determined to have a serious overall risk of bias, as this judgment is inherently applied to non-comparative studies when assessed by Domain 1 (Bias due to confounding) of the ROBINS-I tool.

Although, all 15 studies received a serious risk of bias judgement for Domain 1; all 15 studies were generally well-executed methodologically. This is reflected in judgments of the other domains. Higher risk of bias judgements of other domains were often applied to studies as a result of perioperative events being reported in ways which did not fully align with our primary and secondary outcomes.

Higher risk of bias judgements for Domain 6 (Bias in measurement of outcomes) were primarily applied to studies which reported the CVA diagnostic protocol without mention of radiological imaging, such as CT or MRI scanning, to confirm the diagnosis.

Equally, Domain 7 (Bias in selection of the reported result) was often given a moderate risk of bias as a result of the lack of standardisation in the duration of the postoperative follow up reporting window utilised by studies. Mortality events were often reported as per a 30-day postoperative period, whereas CVA events were reported as per an in-hospital postoperative period. This has the potential for underreporting of CVA events within these cohorts.

The overall serious judgement for all 15 studies, although they were all generally well-executed methodologically, means the overall certainty of the evidence base is likely very low, such that cautious interpretation of our pooled results is required, especially due to the lack of comparative screening strategy data available.

### Study characteristics

The study characteristics of the 15 studies eligible for inclusion in the review are summarised in Table 2. Of these studies, 13 utilised a non-selective screening strategy with the remaining 2 studies utilising a selective screening strategy. The pooled study population demonstrated a high male prevalence, with the proportion of male participants ranging from 69.0% to 86.8% across all cohorts. There was a total of 354 perioperative CVA events across all included studies, with individual perioperative CVA rates ranging from 0.5% to 4.1%.

**Table 2:** Summary of the baseline characteristics of included studies

Study	Study authors and year	Study design	N (total)	Mean age and SD	% Male	Hypertension prevalence	Diabetes mellitus prevalence	Prior CVA/TIA prevalence
1	Ali et al*.* 2020 [22]	Prospective non-randomised clinical trial	360	55 ± 12	83.1% (299/360)	50.0% (180/360)	43.6% (157/360)	7.2% (26/360)
2	Kara 2019 [23]	Retrospective cohort study	166	64 ± 10	78.3% (130/166)	75.9% (126/166)	39.2% (65/166)	4.2% (7/166)
3	Narayan et al*.* 2017 [24]	Prospective cohort study	4364	10.4% > age 65 (454/4364)	NR	70.2% (3063/4364)	45.6% (1988/4364)	2.0% (87/4364)
4	Luchowski et al*.* 2015 [25]	Prospective cohort study	175	66 ± 8	72.6% (127/175)	82.9% (145/175)	29.7% (52/175)	NR
5	Cornily et al*.* 2011 [26]	Retrospective cohort study	205	NR	86.8% (178/205)	54.1% (111/205)	20.0% (41/205)	5.4% (11/205)
6	Kougias et al*.* 2007 [27]	Retrospective cohort study	8277	70 (SD NR)	NR	65.9% (5455/8277)	10.3% (854/8277)	4.9% (403/8277)
7	Durand et al*.* 2004 [28]	Retrospective cohort study	1138	65 ± 11	71.8% (817/1138)	72.5% (825/1138)	32.3% (368/1138)	11.0% (125/1138)
8	Zacharias et al*.* 2002 [29]	Retrospective cohort study	3491	64 ± 10	69.0% (2410/3491)	81.8% (2857/3491)	33.1% (1156/3491)	8.5% (295/3491)
9	Suematsu et al*.* 2000 [30]	Retrospective cohort study	146	64 ± 11	77.4% (113/146)	53.4% (78/146)	39.0% (57/146)	8.2% (12/146)
10	Hirotani et al*.* 2000 [31]	Prospective cohort study	472	64 ± 9	78.0% (368/472)	57.8% (273/472)	49.2% (232/472)	35.4% (167/472)
11	Bilfinger et al*.* 2000 [32]	Prospective cohort study	2071	64 (SD NR)	73.4% (1520/2071)	21.5% (445/2071)	27.3% (566/2071)	6.0% (125/2071)
12	Gaudino et al*.* 1999 [33]	Prospective cohort study	2326	64 ± 8	82.0% (1908/2326)	20.3% (473/2326)	20.9% (485/2326)	13.8% (320/2326)
13	Birincioğlu et al*.* 1999 [34]	Prospective cohort study	722	12.9% > age 60 (93/722)	85.0% (614/722)	26.6% (192/722)	16.5% (119/722)	4.7% (34/722)
14	Mickleborough et al*.* 1996 [35]	Prospective cohort study	1631	60 ± 9	76.3% (1244/1631)	44.1% (720/1631)	21.5% (350/1631)	8.2% (134/1631)
15	Salasidis et al*.* 1995 [36]	Retrospective cohort study	387	63 ± 10	81.4% (315/387)	55.0% (213/387)	24.0% (93/387)	6.5% (25/387)

SD: standard deviation; NR: not reported; CVA: cerebrovascular accident; TIA: transient ischaemic attack

There was considerable heterogeneity in the identified studies with regards to the screening criteria and ECAD thresholds required to trigger pre-defined clinical pathways, Tables 3 and 4. Although most studies utilised unilateral ECAD thresholds between 70 to 80%, these thresholds were often considered differently depending on whether patients had symptomatic or asymptomatic ECAD. Equally, some studies such as Suematsu et al*.* utilised much higher ECAD thresholds (unilateral ECAD > 90%).

**Table 3:** Summary of the 13 included studies utilising non-selective screening and treatment strategies

Study authors	Total screened (N)	Required ECAD thresholds for triggering a pre-defined clinical pathway	Intervention resulting from pre-defined clinical pathway triggered by positive screening result
Ali et al	360	• Unilateral ECAD ≥ 70%	Simultaneous CABG + CEA (via specialist referral)
Kara	166	• Unilateral symptomatic ECAD > 50% • Unilateral asymptomatic ECAD > 80% • Bilateral asymptomatic ECAD > 70%	Simultaneous CEA + CABG (via specialist referral)
Narayan et al	4364	• Unilateral symptomatic ECAD > 70%	Staged CAS + CABG (via specialist referral)
Luchowski et al	175	• Unilateral ECAD ≥ 70%	Staged CAS + CABG
Cornily et al	205	• Unilateral ECAD ≥ 70%	Simultaneous CEA + CABG or staged CEA + CABG (via specialist referral)
Kougias et al	8277	• Unilateral ECAD > 70%	Simultaneous CEA + CABG or simultaneous CEA + CABG (dominant lesion) followed by CEA 4–6 weeks later (contralateral lesion)(both via specialist referral)
Durand et al	1138	•Unilateral ECAD ≥ 70%	Simultaneous CEA + CABG or staged CEA + CABG (both via specialist referral)
Suematsu et al	146	• Unilateral ECAD ≥ 90% • Identification of bilateral ECAD	Simultaneous CEA + CABG (via specialist referral)
Hirotani et al	472	• Unilateral symptomatic ECAD > 75%	Staged CEA + CABG
Bilfinger et al	2071	• Unilateral ECAD ≥ 80%	Simultaneous CEA + CABG
Gaudino et al	2326	• Unilateral ECAD ≥ 80%	Simultaneous CEA + CABG or staged CEA + CABG
Birincioğlu et al	722	• Unilateral ECAD ≥ 80%	Staged CEA + CABG
Salasidis et al	387	• Unilateral ECAD ≥ 80%	Simultaneous CEA + CABG (via specialist referral)

For studies indicating carotid interventions “via specialist referral”, a positive result initiated a formal consultation with a specialist to determine the clinical appropriateness and timing of carotid intervention on a case-by-case basis

CAS: carotid artery stenting; CEA: carotid endarterectomy; CABG: coronary artery bypass surgery; ECAD: extracranial carotid artery disease

**Table 4:** Summary of the two included studies utilising selective screening and treatment strategies

Study authors	Total screened (N)	Indications for patients to undergo screening	Required ECAD thresholds for triggering a pre-defined clinical pathway	Intervention resulting from pre-defined clinical pathway triggered by positive screening result
Zacharias et al	NR	• Prior history or symptoms of CVD • Aged over 60 • Presence of carotid bruits upon examination • Presence of Severe (> 70%) left main coronary artery disease	• Unilateral ECAD > 75%	Simultaneous CEA + CABG
Mickleborough et al	170/1631	• Prior history or symptoms of stroke or unexplained neurological symptoms • Prior history of carotid disease • Presence of carotid bruits or non-palpable carotid pulse upon examination	• Unilateral symptomatic ECAD > 70% • Bilateral asymptomatic ECAD > 70%	Simultaneous CEA + CABG (via specialist referral)

For studies indicating carotid interventions “via specialist referral”, a positive result initiated a formal consultation with a specialist to determine the clinical appropriateness and timing of carotid intervention on a case-by-case basis

NR: not reported; CVD: cerebrovascular disease; CEA: carotid endarterectomy; CABG: coronary artery bypass surgery; ECAD: extracranial carotid artery disease

Heterogeneity between the management of patients with positive screening results also existed between studies, with the intervention most commonly linked with a positive screening result being simultaneous CEA and CABG.

### Primary outcome

Odds ratios were not calculated to compare the neurological outcomes of patients exposed to non-selective and selective screening strategies due to a lack of comparative studies. As a result, our primary outcome was reported as an exploratory pooled perioperative CVA rate for the non-selective screening strategy, alongside the individual study specific perioperative CVA rates for the two studies which utilised a selective screening strategy.

**Fig. 3:**
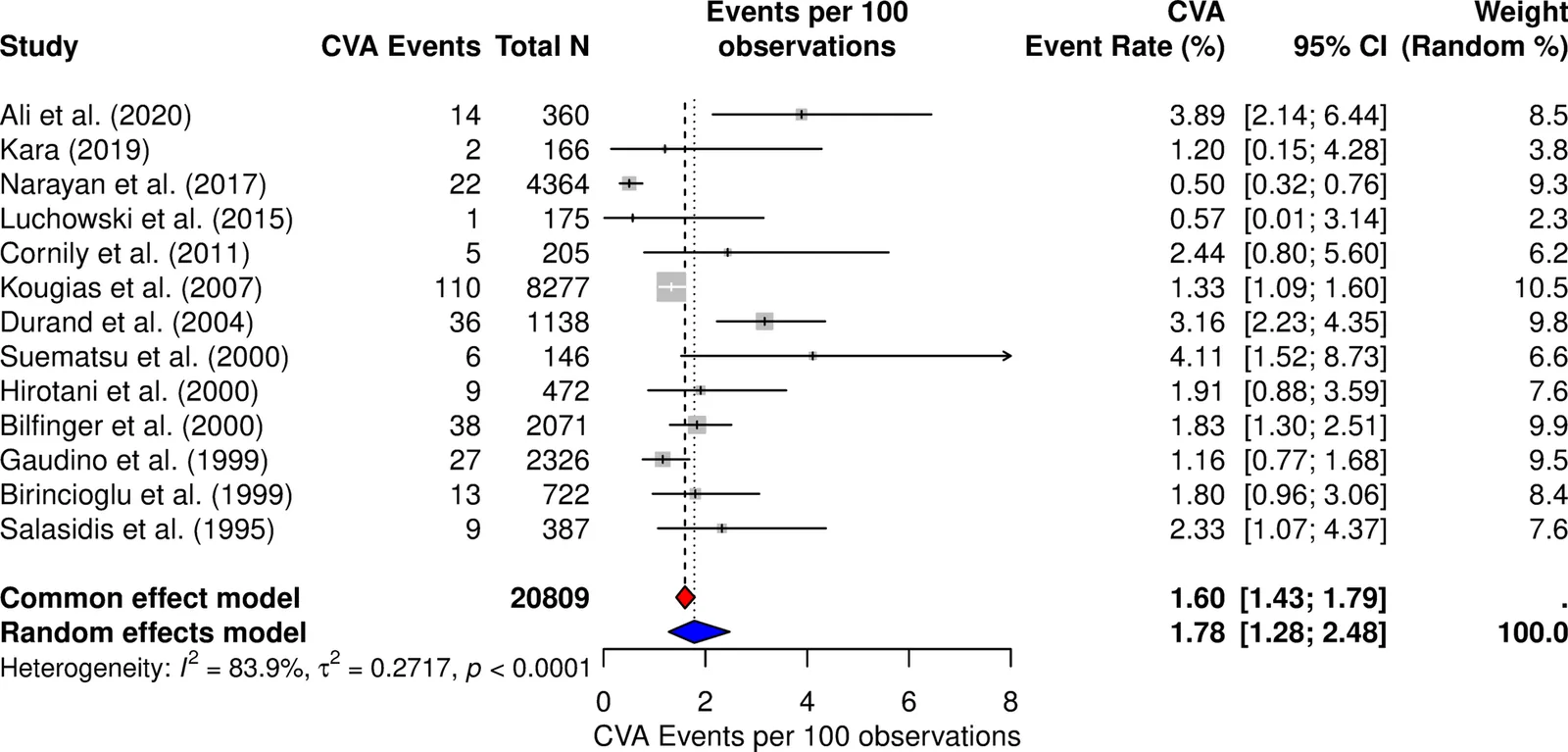
Primary exploratory meta-analysis of perioperative CVA rates of the 13 studies of the non-selective screening cohort. CI: confidence interval; *I* ^2^: the I-squared statistic; τ^2^: the tau-squared statistic

The primary meta-analysis of the 13 included studies of the non-selective cohort, Fig. 3, showed a very high heterogeneity (*I* ^2^ = 83.9%), with a pooled perioperative CVA rate of 1.78% (95% CI: [1.28;2.48]: n = 13, *N* = 20,809). Separately, the two studies of the selective screening cohort reported perioperative CVA rates of 1.16% and 1.23%.

The high heterogeneity of the pooled non-selective screening cohort was explored by a leave-one-out sensitivity analysis, Supplementary Material Figure S1. This identified Narayan et al*.* as a significant contributor to heterogeneity with its exclusion causing a considerable decrease in heterogeneity within the non-selective screening cohort (*I* ^2^ = 74.1%). The sensitivity analysis also confirmed the stability of the pooled perioperative CVA rate for the non-selective screening cohort with this rate ranging between 1.65% and 2.02%. Equally, the assessment of publication bias within this cohort via Egger’s test and funnel plot inspection identified no statistically significant evidence of publication bias. The Egger’s test yielded a p-value of 0.5105 (bias intercept = 0.94, SE = 1.38; t = 0.68, df = 11), with visual inspection of the funnel plot, Supplementary Material Figure S2, also confirming a generally symmetrical distribution of the 13 studies around the pooled estimate.

### Secondary outcomes

The secondary outcomes, such as all-cause mortality rates and complications relating to interventions of the clinical pathway triggered upon identification of a positive screening result are summarised in Table 5, alongside the primary outcome of perioperative CVA rates.

**Table 5:** Summary of clinical and perioperative complications across the 15 included studies

Study authors	All-cause mortality event rate	Perioperative CVA rate of entire cohort	Intervention resulting from pre-defined clinical pathway triggered by positive screening result	Number of patients undergoing Intervention	Major perioperative complications resulting from intervention
Ali et al	NR	3.9% (14/360)	Simultaneous CABG + CEA	4	NR
Kara	NR	1.2% (2/166)	Simultaneous CEA + CABG	5	NR
Narayan et al	2.4% (103/4364)	0.5% (22/4364)	Staged CAS + CABG	9	No major complications
Luchowski et al	0.6% (1/175)	0.6% (1/175) (CVA caused mortality)	Staged CAS + CABG	19	No major complications
Cornily et al	1.5% (3/205)	2.4% (5/205)	Simultaneous CEA + CABG or staged CEA + CABG	5	1 CVA event
Kougias et al	1.8% (146/8277)	1.3% (110/8277)	Simultaneous CEA + CABG or simultaneous CEA + CABG followed by contralateral CEA 4–6 weeks later	277	8 CVA events and 10 deaths
Durand et al	1.9% (22/1138)	3.2% (36/1138)	Simultaneous CEA + CABG or staged CEA + CABG	46	1 CVA event
Zacharias et al	2.0% (71/3491)	1.2% (43/3491)	Simultaneous CEA + CABG	189	2 CVA events and 5 deaths
Suematsu et al	0.7% (1/146)	4.1% (6/146)	Simultaneous CEA + CABG	3	1 death
Hirotani et al	1.7% (8/472)	1.9% (9/472)	Staged CEA + CABG	7	No major complications
Bilfinger et al	2.2% (46/2071)	1.8% (38/2071)	Simultaneous CEA + CABG	84	4 CVA events and 5 deaths
Gaudino et al	1.5% (36/2326)	1.2% (27/2326)	Simultaneous CEA + CABG or staged CEA + CABG	196	2 CVA events and 5 deaths
Birincioğlu et al	4.0% (29/722)	1.8% (13/722)	Staged CEA + CABG	30	No major complications
Mickleborough et al	1.2% (20/1631)	1.2% (19/1631)	Simultaneous CEA + CABG	21	3 CVA events
Salasidis et al	NR	2.3% (9/387)	Simultaneous CEA + CABG	11	No major complications

NR: not reported; CVA: cerebrovascular accident; CAS: carotid artery stenting; CEA: carotid endarterectomy; CABG: coronary artery bypass surgery

Across all 15 studies CU screening resulted in a change to the clinical management of a minority of patients, with the number of patients undergoing carotid interventions as a result of their screening results ranging from 3 patients in smaller cohorts to 277 patients in larger cohorts. In patients who did undergo interventions as a result of positive screening results, the incidence of major complications remained low across screening strategies, with five of the included studies reporting no major intervention-related complications. All-cause mortality rates for the entire populations of each study varied considerably between studies, however, there was no clear pattern between screening strategies.

## Discussion

This systematic review and meta-analysis of 15 studies (N = 25,931) is the first to explore reported neurological outcomes within populations exposed to either non-selective or selective screening strategies. Our findings provide a descriptive synthesis of the current literature which may be utilised by clinicians encountering this scenario. Although the structural limitations of the existing literature prevented a direct comparative synthesis, the low absolute event rates of 1.16% and 1.23% within the selective screening cohorts suggest this targeted approach remains a viable clinical pathway in high-risk patients. These findings may suggest that asymptomatic ECAD cases identified through non-selective screening are not the dominant contributor to perioperative CVA risk, although this interpretation is speculative given the non-comparative nature of the included studies. Furthermore, the clinical utility of any preoperative screening strategy must appropriately balance CVA risk mitigation against the inherent harms of downstream interventions which are a part of the downstream cascade. As non-selective screening inherently leads to a greater number of downstream interventions, its true benefit cannot be directly isolated from the procedural complications associated with these interventions, as this strategy is at a greater risk of exposing patients with ECAD, which may have otherwise been clinically silent, to intervention-related complications.

Equally, when interpreting perioperative CVA rates the multifactorial aetiology of perioperative CVA following CABG must be acknowledged. The clinical utility of preoperative CU screening alone is inherently limited with regards to CVA prevention as the majority of perioperative CVAs occur as a result of aortic emboli, hypoperfusion and atrial fibrillation as opposed to ECAD [10]. As a result, the observed CVA rates cannot be solely attributed to the presence of ECAD or the success or failure of preoperative CU screening cascades alone. Although CU screening can only mitigate a specific subset of strokes, preventing even a fraction of perioperative strokes remains vital, as such preoperative CU screening remains an essential and rapid non-invasive component of a comprehensive preoperative workup providing essential data for overall risk stratification.

A key limitation of this analysis is that all included studies were single-arm, non-comparative cohorts. Although results were stratified by screening strategy, this does not permit comparison between selective and non-selective approaches. Pooled outcomes are highly susceptible to confounding from patient selection, institutional protocols, and procedural variability, therefore, these findings should not be interpreted as evidence of comparative effectiveness but as exploratory analyses. Equally, although the selective screening cohort is small in comparison to the non-selective screening cohort it does represent the best available evidence to date. The lack of studies utilising a selective screening strategy is noteworthy, as this strategy, as opposed to non-selective screening, is the practice advised by current guidelines [8, 11–14]. This may highlight a lack of adherence by surgical centres to current guidelines, with non-selective screening being the default practice of many cardiac centres [37]. This may be the result of many surgical centres struggling with the operational logistics required to strictly enforce a risk-stratified selective screening pathway, as such centres may adopt a uniform non-selective approach for the sake of simplicity. Equally, some centres may utilise non-selective screening strategies, despite the lack of supportive comparative data, in order to avoid missing asymptomatic but severe ECAD lesions in patients scheduled to undergo CABG.

A primary finding of this review was the inherent methodological limitation of the existing literature. All 15 studies were categorised as having overall serious risk of bias judgements, primarily because they were single-arm, non-comparative cohorts with regards to the preoperative CU screening strategies utilised which limits the ability of the meta-analysis to provide definitive conclusions and instead positions it as an exploratory analysis. Equally, lack of a standardised 30-day postoperative follow up period exhibited by many studies has the potential for underreporting of perioperative CVA events.

Despite these limitations, the stability of the results sensitivity analysis of the non-selective cohort across diverse populations may be consistent with current guideline recommendations with regards to perioperative neurological outcomes [8, 11–14]. The decrease in heterogeneity observed during the exclusion of Narayan et al*.* during the sensitivity analysis is likely a result of this cohort having the lowest prevalence of prior CVA/TIA history of all included studies. Prior CVA/TIA history is a strong predictor of subsequent neurological events and as such would explain the lower CVA event rates of the Narayan et al*.* cohort [38].

While the results of the funnel plot inspection and Egger’s test did not identify statistically significant evidence of publication bias within the non-selective screening cohort, the presence of institutional reporting bias must be considered. Cardiac centres with highly favourable clinical outcomes, such as low perioperative CVA rates, are more inclined to publish studies relating to screening and outcomes. As a result, this has the potential to underrepresent real-world averages.

Another key consideration when interpreting the event rates within this review is the significant change in the natural history of asymptomatic ECAD throughout the period covered by this review (1995–2025). Changes in medical therapy have significantly reduced baseline CVA risks compared to some of the historical cohorts included in this review. Recent data estimates that nearly 70% of individuals with high-grade ECAD (75% to 99%) will remain asymptomatic and free from CVA over five years; these figures differ noticeably from reports of the 1990s and early 2000s [39]. As a result, the pooled rates of this review should be interpreted with caution when applied to current clinical practice.

A further limitation of the literature was the lack of standardisation in the ECAD thresholds used to define a positive screening result, with studies such as Suematsu et al*.* utilising much higher ECAD thresholds (unilateral ECAD > 90%) than all other included studies. The standardisation of these thresholds is essential to the success of any screening strategy regardless of whether it is selective or non-selective in nature. Equally, the lack of standardisation prevents the ability to perform a robust investigation into the underlying sources of clinical heterogeneity.

Zhu et al*.* demonstrated CU screening in populations with 10-year atherosclerotic cardiovascular disease (ASCVD) risk scores exceeding 30%, yields a cost-effective incremental cost-effective ratio (ICER) [40]. This suggests that selective screening strategies may be more resource-efficient in high-risk populations; however, this review did not directly assess cost-effectiveness.

Overall, our findings provide a descriptive context for surgical teams navigating the current guidelines. Although the non-comparable nature of the existing literature prevents a direct comparison between the two screening strategies, the studies of the selective screening cohort (n = 2) documented low absolute perioperative stroke rates of 1.16% and 1.23%. Independently, across the cohorts evaluating a non-selective screening strategy (n = 13), the overall pooled perioperative stroke rate was 1.78%. Equally, the small number of studies within the selective screening cohort identifies a current reliance of surgical teams on non-selective screening strategies. This highlights a critical evidence gap, as a result, future research should aim to fill this gap, preferably through a large scale RCT aiming to compare the two screening strategies, despite potential ethical issues in trial design. Until then, our data support the continued practice of selective screening as an effective strategy in the isolated CABG population.

### Limitations

Although this was the first meta-analysis of preoperative CU screening strategies for CABG, there were several key limitations.

Our definition of selective screening as per the methods section is inherently broad and combines heterogeneous clinical indications which, as a result, introduces within-group heterogeneity. However, as the existing literature lacks the adequate patient-level data needed to isolate outcomes for individual risk factors our findings must be interpreted as capturing the results of real-world practice as opposed to evaluating the efficacy of a single, standardised selective screening strategy.

The selective screening cohort (n = 2) was significantly smaller than the non-selective screening cohort (n = 13). This limits the statistical power of the results to detect small differences despite a substantial selective screening cohort population (Total *N* = 5122).

Critically, all 15 included studies were non-comparative studies. This introduces potential selection bias and confounding as the screening strategies a patient underwent were dictated by institutional protocols as opposed to random allocation.

Additionally, although we performed a sensitivity analysis to identify significant contributors to heterogeneity, such as Narayan et al*.*, we did not control for variations such as differences in CABG technique between studies. However, it should be noted that the CORONARY trial identified there to be no significant difference in CVA incidence between groups of patients undergoing either on-pump or off-pump CABG [41]. Equally, with regards to exploring heterogeneity a further limitation of the literature was both the lack of standardisation in ECAD thresholds used to define a positive screening result, as well as the pooling of outcome data in studies such as Cornily et al. and Durand et al. whereby although patients could undergo either simultaneous or staged interventions outcome data was only available in a pooled format for the entire cohort. This lack of standardisation of ECAD thresholds as well as pooling of outcome data prevents the ability to perform a robust quantitative investigation into the underlying sources of clinical heterogeneity.

Similarly, another key limitation of the existing literature with regards to our secondary outcomes is the inability to determine the precise chronological timing of major postoperative complications in patients undergoing staged interventions, as the majority of included studies utilising staged management strategies report major complications as a single cumulative perioperative outcome which encompasses the entire management strategy.

Finally, as screening cascades inherently couple diagnostic strategy with downstream management strategies, the variability in the given management strategies utilised in different studies (e.g. simultaneous CEA + CABG versus staged CAS + CABG) represents a major confounder. As a result, the observed pooled perioperative CVA rate of the non-selective cohort partially reflects the impact of these non-standardised screening cascades in preventing perioperative CVA as opposed to the independent efficacy of the screening strategy alone, although these rates are ultimately confounded by the multifactorial pathophysiology of postoperative CVA.

## Conclusion

This review provides a descriptive synthesis of perioperative CVA rates across cohorts exposed to either selective or non-selective preoperative CU screening prior to non-emergency isolated CABG and does not establish comparative effectiveness. Although the low absolute event rates observed within the selective screening cohort aligns with current guideline recommendations, the structural limitations and disproportion in the number of available studies in the existing literature prevent definitive comparison. However, the existing data does provide a level of reassurance for the continued practice of selective screening as advised by current guidelines. These findings highlight the need for standardised ECAD thresholds across screening strategies, as well as further high-quality research, such as RCTs, directly comparing the effectiveness of each screening strategy.

## Supplementary Information

Additional file 1 Leave-one-out sensitivity analysis of all 13 studies of the non-selective screening cohort. Abbreviations: CI: confidence interval; *I *^2^: the I-squared statistic.

Additional file 2 Funnel plot of all 13 studies of the non-selective screening cohort. The Egger’s test yielded a p-value of 0.5105 (bias intercept = 0.94, SE = 1.38; t = 0.68, df = 11). Abbreviations: SE: standard error; t: the t statistic; df: degrees of freedom.

Additional file 3.

Additional file 4.

## Data Availability

The datasets supporting the conclusions of this article are included within the article and its additional files.

## Abbreviations

CABG: Coronary artery bypass grafting
CAS: Carotid artery stenting
CEA: Carotid endarterectomy
CHD: Coronary heart disease
CI: Confidence interval
CT: Computed tomography
CU: Carotid ultrasound
CVA: Cerebrovascular accident
ECAD: Extracranial carotid artery disease
MI: Myocardial infarction
MRI: Magnetic resonance imaging
RCT: Randomised controlled trial
ROBINS-I: Risk of bias in non-randomised studies of interventions
TIA: Transient ischaemic attack
