# Supplementary material for: Selective and non-selective carotid ultrasound screening and perioperative stroke incidence in the coronary artery bypass grafting population: a systematic review and meta-analysis
Source: J Cardiothorac Surg. 2026 Jul 19;21:792. doi: 10.1186/s13019-026-04616-6 (PMC13662987; doi:10.1186/s13019-026-04616-6)
Supplement: Supplementary file 1 [file 13019_2026_4616_MOESM1_ESM.pdf]

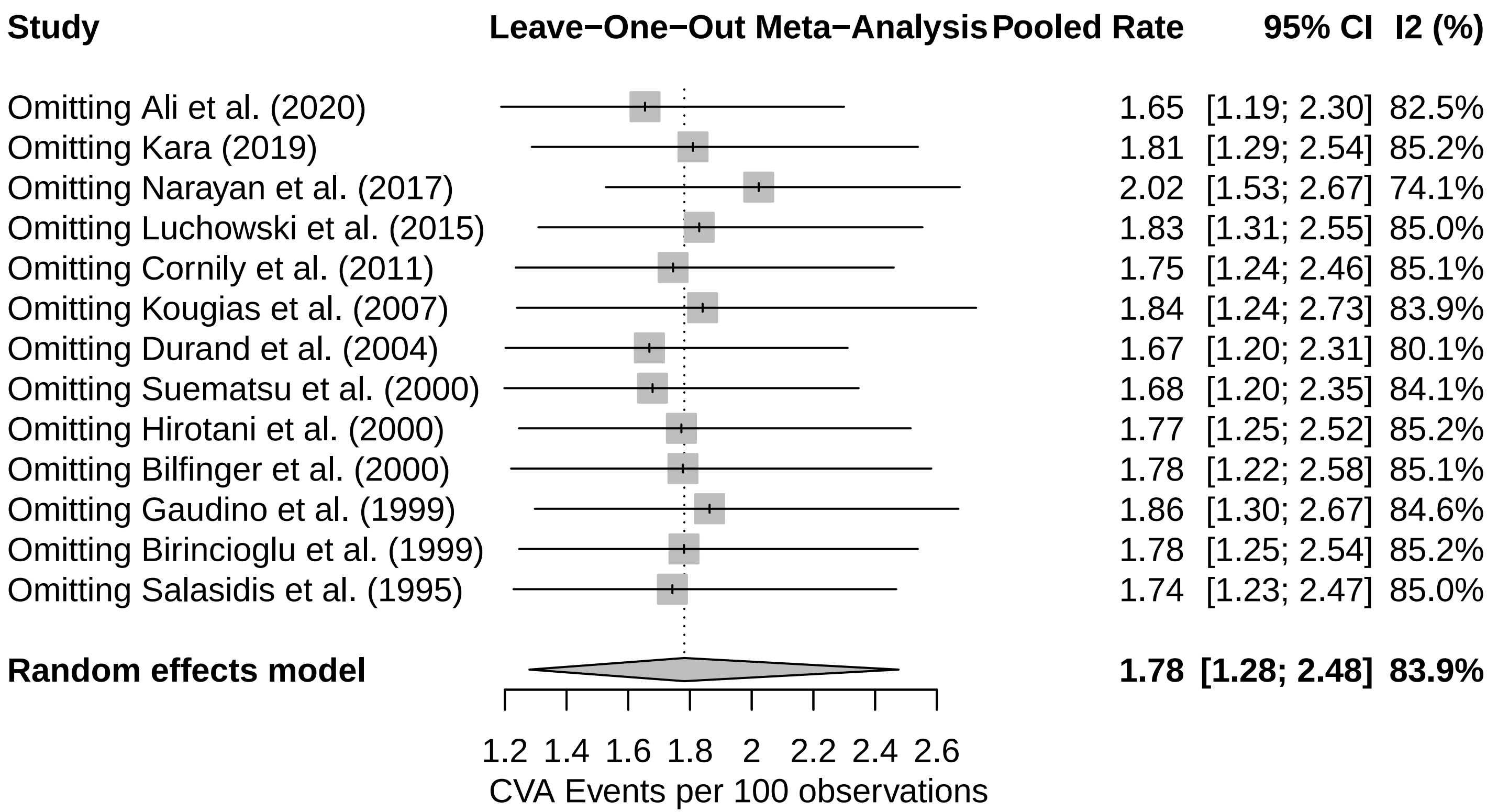
Study
Leave−One−Out Meta−Analysis
Pooled Rate
95% CI
I2 (%)
Omitting Ali et al. (2020) 1.65 [1.19; 2.30] 82.5%
Omitting Kara (2019) 1.81 [1.29; 2.54] 85.2%
Omitting Narayan et al. (2017) 2.02 [1.53; 2.67] 74.1%
Omitting Luchowski et al. (2015) 1.83 [1.31; 2.55] 85.0%
Omitting Cornily et al. (2011) 1.75 [1.24; 2.46] 85.1%
Omitting Kougias et al. (2007) 1.84 [1.24; 2.73] 83.9%
Omitting Durand et al. (2004) 1.67 [1.20; 2.31] 80.1%
Omitting Suematsu et al. (2000) 1.68 [1.20; 2.35] 84.1%
Omitting Hirotani et al. (2000) 1.77 [1.25; 2.52] 85.2%
Omitting Bilfinger et al. (2000) 1.78 [1.22; 2.58] 85.1%
Omitting Gaudino et al. (1999) 1.86 [1.30; 2.67] 84.6%
Omitting Birincioglu et al. (1999) 1.78 [1.25; 2.54] 85.2%
Omitting Salasidis et al. (1995) 1.74 [1.23; 2.47] 85.0%
Random effects model 1.78 [1.28; 2.48] 83.9%
1.2 1.4 1.6 1.8 2 2.2 2.4 2.6
CVA Events per 100 observations
