# Supplementary material for: Selective and non-selective carotid ultrasound screening and perioperative stroke incidence in the coronary artery bypass grafting population: a systematic review and meta-analysis
Source: J Cardiothorac Surg. 2026 Jul 19;21:792. doi: 10.1186/s13019-026-04616-6 (PMC13662987; doi:10.1186/s13019-026-04616-6)
Supplement: Supplementary file 2 [file 13019_2026_4616_MOESM2_ESM.pdf]

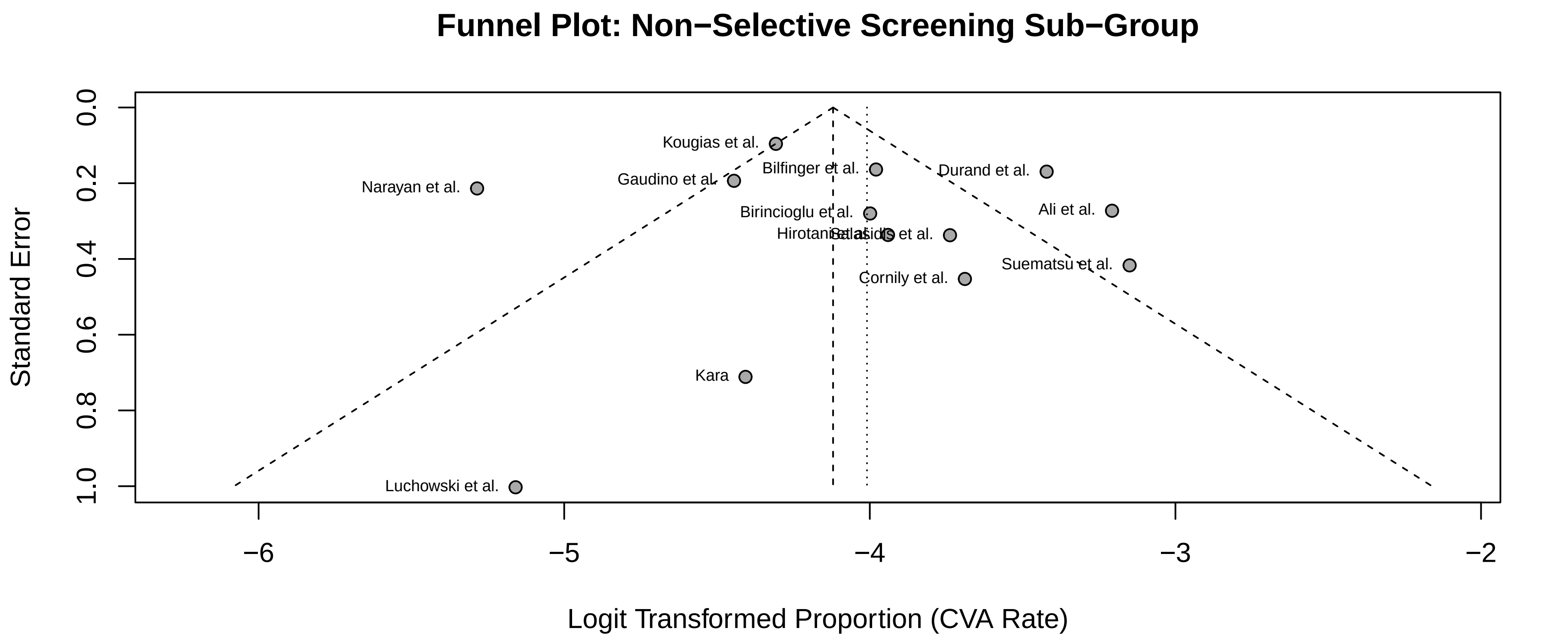

Funnel Plot: Non−Selective Screening Sub−Group
Standard Error
0.0
0.2
0.4
0.6
0.8
1.0
Kougias et al.
Bilfinger et al.
Durand et al.
Narayan et al.
Gaudino et al.
Birincioglu et al.
Ali et al.
Hirotani et al.
Selasidis et al.
Suematsu et al.
Cornily et al.
Kara
Luchowski et al.
−6
−5
−4
−3
−2
Logit Transformed Proportion (CVA Rate)
