# Supplementary material for: Selective and non-selective carotid ultrasound screening and perioperative stroke incidence in the coronary artery bypass grafting population: a systematic review and meta-analysis
Source: J Cardiothorac Surg. 2026 Jul 19;21:792. doi: 10.1186/s13019-026-04616-6 (PMC13662987; doi:10.1186/s13019-026-04616-6)
Supplement: Supplementary file 3 [file 13019_2026_4616_MOESM3_ESM.docx]

1. **Ali *et al.* 2020**

**Study type:** Prospective non-randomised clinical trial

**Participants:** Patients scheduled for isolated CABG

**Intervention group:** Non-selective screening group (n=360)

**Comparison group:** Selective screening group as per our target RCT

**Outcomes assessed**

- Outcome 1 – Perioperative CVA incidence within the 30-day postoperative period

| Outcome | D1: bias due to confounding | D2: bias in selection of participants | D3: bias in classification of intervention | D4: bias due to deviations from intended intervention | D5: bias due to missing outcome data | D6: bias in measurement of outcome | D7: bias in selection of the reported result | Overall risk of bias judgment for outcome |
| --- | --- | --- | --- | --- | --- | --- | --- | --- |
| Outcome 1 | Serious^1^ | Low^2^ | Low^3^ | Low^4^ | Low^5^ | Low^6^ | Moderate^7^ | Serious |

**Reasons for judgements of each domain**

^1^The study lacks a selective screening comparator group, as well as this confounding domains such as aortic calcification were not reported. Equally, the reported perioperative CVA event count did not appear to have been statistically adjusted for confounding factors. However, the authors did perform logistic regression analyses to identify predictors of neurological complications

^2^All participants were enrolled for non-selective preoperative CU screening as per the institutions protocol for patients scheduled to undergo elective CABG

^3^The non-selective screening group was clearly defined by the institutions protocol, with all participants being preoperatively assigned to and undergoing screening as per this strategy. As a result, the intervention classification occurred independently of subsequent neurological outcomes

^4^The study followed a pre-defined institutional clinical pathway, with the results of the intervention leading to expected changes in management such as referral for simultaneous CABG and CEA

^5^The study provided data for the entire 360 patient cohort with all patients being followed up as per the 30-day postoperative period. There is no evidence of participants being excluded due to missing a screening result

^6^Although assessors were not blinded to screening results, CVA events were given a pre-defined and standard definition. In this case CVA confirmation via neurological examination and MRI / CT imaging was required for formal diagnosis

^7^The study reported findings for the entire 360-patient cohort. However, there is evidence of selective reporting as one of the secondary endpoints of the study was mortality data which was not reported

2. **Kara 2019**

**Study type:** Retrospective cohort study

**Participants:** Patients scheduled for isolated CABG

**Intervention group:** Non-selective screening group (n=166)

**Comparison group:** Selective screening group as per our target RCT

**Outcomes assessed**

- Outcome 1 – Perioperative CVA incidence within the 30-day postoperative period

| Outcome | D1: bias due to confounding | D2: bias in selection of participants | D3: bias in classification of intervention | D4: bias due to deviations from intended intervention | D5: bias due to missing outcome data | D6: bias in measurement of outcome | D7: bias in selection of the reported result | Overall risk of bias judgment for outcome |
| --- | --- | --- | --- | --- | --- | --- | --- | --- |
| Outcome 1 | Serious^1^ | Low^2^ | Low^3^ | Low^4^ | Low^5^ | Low^6^ | Low^7^ | Serious |

**Reasons for judgements of each domain**

^1^The study lacks a selective screening comparator group, as well as this confounding domains such as aortic calcification were not reported. Equally, the reported perioperative CVA event count did not appear to have been statistically adjusted for confounding factors. However, the authors did perform logistic regression to identify predictors of CAS

^2^All participants were enrolled for non-selective preoperative CU screening as per the institutions protocol for patients scheduled to undergo isolated elective CABG

^3^The non-selective screening group was clearly defined by the institutions protocol, with all participants being preoperatively assigned to and undergoing screening as per this strategy. As a result, the intervention classification occurred independently of subsequent neurological outcomes

^4^The study followed a pre-defined institutional clinical pathway, with the results of the intervention leading to expected changes in management such as referral for simultaneous CEA and CABG

^5^The study provided data for the entire 166 patient cohort with all patients being followed up as per the 30-day postoperative period. There is no evidence of participants being excluded due to missing a screening result

^6^Although assessors were not blinded to screening results, CVA events were given a pre-defined and standard definition. In this case CVA confirmation via MRI imaging was required for formal diagnosis

^7^The study reported findings for the entire 166-patient cohort

3. **Narayan *et al.* 2017**

**Study type:** Prospective cohort study

**Participants:** Patients scheduled for isolated CABG

**Intervention group:** Non-selective screening group (n=4364)

**Comparison group:** Selective screening group as per our target RCT

**Outcomes assessed**

- Outcome 1 – Perioperative mortality within the 30-day postoperative period
- Outcome 2 – Perioperative CVA incidence within the 30-day postoperative period

| Outcome | D1: bias due to confounding | D2: bias in selection of participants | D3: bias in classification of intervention | D4: bias due to deviations from intended intervention | D5: bias due to missing outcome data | D6: bias in measurement of outcome | D7: bias in selection of the reported result | Overall risk of bias judgment for outcome |
| --- | --- | --- | --- | --- | --- | --- | --- | --- |
| Outcome 1 | Serious^1^ | Low^2^ | Low^3^ | Low^4^ | Moderate^5^ | Low^6^ | Moderate^7^ | Serious |
| Outcome 2 | Serious^1^ | Low^2^ | Low^3^ | Low^4^ | Moderate^5^ | Low^6^ | Moderate^7^ | Serious |

**Reasons for judgements of each domain**

^1^The study lacks a selective screening comparator group, as well as this confounding domains such as biological sex proportions of the population and aortic calcification were not reported. Equally, the reported all-cause mortality and perioperative CVA event counts did not appear to have been statistically adjusted for confounding factors. However, the authors did perform multivariate regression to identify predictors of adverse neurological events

^2^All participants were enrolled for non-selective preoperative CU screening as per the institutions protocol for patients scheduled to undergo isolated CABG

^3^The non-selective screening group was clearly defined by the institutions protocol, with all participants being preoperatively assigned to and undergoing screening as per this strategy. As a result, the intervention classification occurred independently of subsequent postoperative outcomes

^4^The study followed a pre-defined institutional clinical pathway, with the results of the intervention leading to expected changes in management such as referral for staged carotid artery stenting and CABG

^5^The study provided data for the entire 4364 patient cohort with all patients being followed up as per the in-hospital postoperative period. However, there was a potential for some patients to be followed up for a period greater than the in-hospital period if concerns about the patient were raised, as such there was a potential for an increased number of CVA events reported. There is no evidence of participants being excluded due to missing a screening result

^6^Although assessors were not blinded to screening results, all-cause mortality events are objective events. Major adverse neurological events (CVAs) were given a pre-defined and standard definition. In this case CVA confirmation via neurological examination and MRI / CT imaging was required for formal diagnosis

^7^The study reported findings for the entire 4364 patient cohort. Differences in the duration of the postoperative period experienced by different patients, depending on whether concerns were raised or not, has the potential for overreporting of CVA events. However, the utilisation of an in-hospital postoperative follow up period as opposed to the standard 30-day postoperative period has the potential for underreporting of CVA events

4. **Luchowski *et al.* 2015**

**Study type:** Prospective cohort study

**Participants:** Patients scheduled for isolated CABG

**Intervention group:** Non-selective screening group (n=175)

**Comparison group:** Selective screening group as per our target RCT

**Outcomes assessed**

- Outcome 1 – Perioperative mortality within the 30-day postoperative period
- Outcome 2 – Perioperative CVA incidence within the 30-day postoperative period

| Outcome | D1: bias due to confounding | D2: bias in selection of participants | D3: bias in classification of intervention | D4: bias due to deviations from intended intervention | D5: bias due to missing outcome data | D6: bias in measurement of outcome | D7: bias in selection of the reported result | Overall risk of bias judgment for outcome |
| --- | --- | --- | --- | --- | --- | --- | --- | --- |
| Outcome 1 | Serious^1^ | Low^2^ | Low^3^ | Low^4^ | Low^5^ | Moderate^6^ | Moderate ^7^ | Serious |
| Outcome 2 | Serious^1^ | Low^2^ | Low^3^ | Low^4^ | Low^5^ | Moderate^6^ | Moderate ^7^ | Serious |

**Reasons for judgements of each domain**

^1^The study lacks a selective screening comparator group, as well as this confounding domains such as prior CVA/TIA history, left main stem disease prevalence, aortic calcification and intra-operative aortic clamp time were not reported. Equally, the reported all-cause mortality and perioperative CVA event counts did not appear to have been statistically adjusted for confounding factors. However, the authors did perform statistical analyses to identify predictors of arterial stenosis

^2^All participants were enrolled for non-selective preoperative CU screening as per the institutions protocol for patients scheduled to undergo elective CABG

^3^The non-selective screening group was clearly defined by the institutions protocol, with all participants being preoperatively assigned to and undergoing screening as per this strategy. As a result, the intervention classification occurred independently of subsequent postoperative outcomes

^4^The study followed a pre-defined institutional clinical pathway, with the results of the intervention leading to expected changes in such as referral for staged carotid artery stenting and CABG. However, the co-intervention of transcranial ultrasound scanning should be acknowledged as having the potential to increase the number of patients exposed to an altered management, e.g. increased attention to haemodynamic management during CABG

^5^The study provided data for the entire 175 patient cohort with all patients being followed up as per the staged 1 week, 6 month and 1 year postoperative period. There is no evidence of participants being excluded due to missing a screening result

^6^Although assessors were not blinded to screening results, all-cause mortality events are objective events. The studies pre-defined definition for CVA events was relatively vague and lacked the requirement of radiological scanning or neurological examination for formal diagnosis

^7^The study reported findings for the entire 175 patient cohort. The utilisation of the 1 year postoperative follow up period has the potential for overreporting of CVA events as this is far longer in duration than the standard 30-day postoperative period

5. **Cornily *et al.* 2011**

**Study type:** Retrospective cohort study

**Participants:** Patients scheduled for first time isolated CABG

**Intervention group:** Non-selective screening group (n=205)

**Comparison group:** Selective screening group as per our target RCT

**Outcomes assessed**

- Outcome 1 – Perioperative mortality within the 30-day postoperative period
- Outcome 2 – Perioperative CVA incidence within the 30-day postoperative period

| Outcome | D1: bias due to confounding | D2: bias in selection of participants | D3: bias in classification of intervention | D4: bias due to deviations from intended intervention | D5: bias due to missing outcome data | D6: bias in measurement of outcome | D7: bias in selection of the reported result | Overall risk of bias judgment for outcome |
| --- | --- | --- | --- | --- | --- | --- | --- | --- |
| Outcome 1 | Serious^1^ | Low^2^ | Low^3^ | Moderate^4^ | Low^5^ | Low^6^ | Moderate^7^ | Serious |
| Outcome 2 | Serious^1^ | Low^2^ | Low^3^ | Moderate^4^ | Low^5^ | Low^6^ | Moderate^7^ | Serious |

**Reasons for judgements of each domain**

^1^The study lacks a selective screening comparator group, as well as this confounding domains such as mean age, left main stem disease prevalence, aortic calcification and intra-operative aortic clamp time were not reported. Equally, the reported all-cause mortality and perioperative CVA event counts did not appear to have been statistically adjusted for confounding factors. However, the authors did perform multivariate logistic regression to identify predictors of stroke

^2^All participants were enrolled for non-selective preoperative CU screening as per the institutions protocol for patients scheduled to undergo isolated CABG

^3^The non-selective screening group was clearly defined by the institutions protocol, with all participants being preoperatively assigned to and undergoing screening as per this strategy. As a result, the intervention classification occurred independently of subsequent postoperative outcomes

^4^The study followed a pre-defined institutional clinical pathway, with the results of the intervention leading to expected changes in management such as referral for simultaneous or staged CEA and CABG. However, it should be noted that the reason patients received simultaneous as opposed to staged CEA and CABG was not reported

^5^The study provided data for the entire 205 patient cohort with all patients being followed up as per the in-hospital postoperative period. There is no evidence of participants being excluded due to missing a screening result

^6^Although assessors were not blinded to screening results, all-cause mortality events are objective events. CVA events were given a pre-defined and standard definition. In this case CVA confirmation via MRI / CT imaging was required for formal diagnosis

^7^The study reported findings for the entire 205 patient cohort. The utilisation of the in-hospital postoperative follow up period has the potential for underreporting CVA events as this is often shorter in duration than the standard 30-day postoperative period

6. **Kougias *et al.* 2007**

**Study type:** Retrospective cohort study

**Participants:** Patients scheduled for isolated CABG

**Intervention group:** Non-selective screening group (n=8277)

**Comparison group:** Selective screening group as per our target RCT

**Outcomes assessed**

- Outcome 1 – Perioperative mortality within the 30-day postoperative period
- Outcome 2 – Perioperative CVA incidence within the 30-day postoperative period

| Outcome | D1: bias due to confounding | D2: bias in selection of participants | D3: bias in classification of intervention | D4: bias due to deviations from intended intervention | D5: bias due to missing outcome data | D6: bias in measurement of outcome | D7: bias in selection of the reported result | Overall risk of bias judgment for outcome |
| --- | --- | --- | --- | --- | --- | --- | --- | --- |
| Outcome 1 | Serious^1^ | Low^2^ | Low^3^ | Low^4^ | Low^5^ | Low^6^ | Low^7^ | Serious |
| Outcome 2 | Serious^1^ | Low^2^ | Low^3^ | Low^4^ | Low^5^ | Low^6^ | Low^7^ | Serious |

**Reasons for judgements of each domain**

^1^The study lacks a selective screening comparator group, as well as this confounding domains such as biological sex, left main stem disease prevalence, aortic calcification and intra-operative aortic clamp time were not reported. Equally, the reported all-cause mortality and perioperative CVA event counts did not appear to have been statistically adjusted for confounding factors. However, the authors did perform statistical analyses to compare subgroups within their cohort

^2^ All participants were enrolled for non-selective preoperative CU screening as per the institutions protocol for patients scheduled to undergo elective CABG

^3^The non-selective screening group was clearly defined by the institutions protocol, with all participants being preoperatively assigned to and undergoing screening as per this strategy. As a result, the intervention classification occurred independently of subsequent postoperative outcomes

^4^The study followed a pre-defined institutional clinical pathway, with the results of the intervention leading to expected changes in management such as referral for simultaneous CEA and CABG or simultaneous CEA and CABG followed by contralateral CEA 4-6 weeks later. The decision for patients to receive multiple carotid interventions was primarily based on the presence of unilateral versus bilateral CAS

^5^The study provided data for the entire 8277 patient cohort with all patients being followed up as per the 30-day postoperative follow up period. There is no evidence of participants being excluded due to missing a screening result

^6^Although assessors were not blinded to screening results, all-cause mortality events are objective events. CVA events although not formally defined did have criteria to be met for formal diagnosis. In this case CVA confirmation via neurological examination and radiographic imaging was required for formal diagnosis

^7^The study reported findings for the entire 8277 patient cohort

7. **Durand *et al.* 2004**

**Study type:** Retrospective cohort study

**Participants:** Patients scheduled for isolated CABG

**Intervention group:** Non-selective screening group (n=1138)

**Comparison group:** Selective screening group as per our target RCT

**Outcomes assessed**

- Outcome 1 – Perioperative mortality within the 30-day postoperative period
- Outcome 2 – Perioperative CVA incidence within the 30-day postoperative period

| Outcome | D1: bias due to confounding | D2: bias in selection of participants | D3: bias in classification of intervention | D4: bias due to deviations from intended intervention | D5: bias due to missing outcome data | D6: bias in measurement of outcome | D7: bias in selection of the reported result | Overall risk of bias judgment for outcome |
| --- | --- | --- | --- | --- | --- | --- | --- | --- |
| Outcome 1 | Serious^1^ | Low^2^ | Low^3^ | Moderate^4^ | Low^5^ | Low^6^ | Moderate^7^ | Serious |
| Outcome 2 | Serious^1^ | Low^2^ | Low^3^ | Moderate^4^ | Low^5^ | Low^6^ | Moderate^7^ | Serious |

**Reasons for judgements of each domain**

^1^The study lacks a selective screening comparator group, as well as this confounding domains such as aortic calcification and intra-operative aortic clamp time were not reported. Equally, the reported all-cause mortality and perioperative CVA event counts did not appear to have been statistically adjusted for confounding factors. However, the authors did perform multiple logistic regression analysis to identify predictors of significant CAS

^2^ All participants were enrolled for non-selective preoperative CU screening as per the institutions protocol for patients scheduled to undergo isolated CABG

^3^The non-selective screening group was clearly defined by the institutions protocol, with all participants being preoperatively assigned to and undergoing screening as per this strategy, as a result the intervention classification occurred independently of subsequent adverse postoperative outcomes

^4^The study followed a pre-defined institutional clinical pathway, with the results of the intervention leading to expected changes in management such as referral for simultaneous or staged CEA and CABG. However, it should be noted the reason patients received simultaneous as opposed to staged CEA was not reported

^5^The study provided data for the entire 1138 patient cohort with all patients being followed up as per the in-hospital postoperative follow up period. There is no evidence of participants being excluded due to missing a screening result

^6^Although assessors were not blinded to screening results, all-cause mortality events are objective events. CVA events were given a pre-defined and standard definition. In this case CVA confirmation via neurological examination and CT / MRI imaging was required for formal diagnosis

^7^The study reported findings for the entire 1138 patient cohort. There is potential for underreporting of CVA events as a result of the utilisation of the in-hospital postoperative follow up period likely often being shorter than the standard 30-day postoperative period

8. **Zacharias *et al.* 2002**

**Study type:** Retrospective cohort study

**Participants:** Patients scheduled for isolated CABG

**Intervention group:** Non-selective screening group as per our target RCT

**Comparison group:** Selective screening group (n=3491)

**Outcomes assessed**

- Outcome 1 – Perioperative mortality within the 30-day postoperative period
- Outcome 2 – Perioperative CVA incidence within the 30-day postoperative period

| Outcome | D1: bias due to confounding | D2: bias in selection of participants | D3: bias in classification of intervention | D4: bias due to deviations from intended intervention | D5: bias due to missing outcome data | D6: bias in measurement of outcome | D7: bias in selection of the reported result | Overall risk of bias judgment for outcome |
| --- | --- | --- | --- | --- | --- | --- | --- | --- |
| Outcome 1 | Serious^1^ | Low^2^ | Low^3^ | Low^4^ | Low^5^ | Moderate^6^ | Moderate ^7^ | Serious |
| Outcome 2 | Serious^1^ | Low^2^ | Low^3^ | Low^4^ | Low^5^ | Moderate^6^ | Moderate ^7^ | Serious |

**Reasons for judgements of each domain**

^1^The study lacks a non-selective screening comparator group, as well as this confounding domains such as aortic calcification were not reported. Equally, the reported all-cause mortality and perioperative CVA event counts did not appear to have been statistically adjusted for confounding factors. However, the authors did perform survival analyses for sub-groups within the cohort

^2^All participants were enrolled for selective preoperative CU screening as per the institutions protocol for patients scheduled to undergo non-emergency CABG

^3^The selective screening group was clearly defined by the institutions protocol, primarily targeting patients with; prior CVD history, over age 60, carotid bruit or severe left main stem disease. All participants were preoperatively assigned to undergo screening as per this strategy, as a result the intervention classification occurred independently of subsequent neurological outcomes

^4^The study followed a pre-defined institutional clinical pathway, with the results of the intervention leading to expected changes in management such as referral for simultaneous CEA and CABG

^5^The study provided data for the entire 3491 patient cohort with all patients being followed up as per a 30-day postoperative period for all-cause mortality and an in-hospital postoperative period for CVA. There is no evidence of participants being excluded due to missing a screening result

^6^Assessors were not blinded to screening results. CVA, although not formally defined did have criteria to be met for formal diagnosis. However, in this case only required diagnosis of CVA via neurology staff as opposed to requiring radiological imaging for confirmation

^7^The study reported findings for the entire 3491 patient cohort. There is potential for underreporting of CVA events as a result of the utilisation of the in-hospital postoperative follow up period likely often being shorter than the standard 30-day postoperative period

9. **Suematsu *et al.* 2000**

**Study type:** Prospective cohort study

**Participants:** Patients scheduled for isolated CABG

**Intervention group:** Non-selective screening group (n=146)

**Comparison group:** Selective screening group as per our target RCT

**Outcomes assessed**

- Outcome 1 – Perioperative mortality within the 30-day postoperative period
- Outcome 2 – Perioperative CVA incidence within the 30-day postoperative period

| Outcome | D1: bias due to confounding | D2: bias in selection of participants | D3: bias in classification of intervention | D4: bias due to deviations from intended intervention | D5: bias due to missing outcome data | D6: bias in measurement of outcome | D7: bias in selection of the reported result | Overall risk of bias judgment for outcome |
| --- | --- | --- | --- | --- | --- | --- | --- | --- |
| Outcome 1 | Serious^1^ | Low^2^ | Low^3^ | Low^4^ | Low^5^ | Low^6^ | Moderate^7^ | Serious |
| Outcome 2 | Serious^1^ | Low^2^ | Low^3^ | Low^4^ | Low^5^ | Low^6^ | Moderate^7^ | Serious |

**Reasons for judgements of each domain**

^1^The study lacks a selective screening comparator group, as well as this confounding domains such as aortic calcification, left main stem disease prevalence, peripheral arterial disease prevalence and intra-operative aortic clamp time were not reported. Equally, the reported all-cause mortality and perioperative CVA event counts did not appear to have been statistically adjusted for confounding factors. However, the authors did perform multivariate analyses to identify predictors of abnormal CU results

^2^All participants were enrolled for non-selective preoperative CU screening as per the institutions protocol for patients scheduled to undergo isolated CABG

^3^The non-selective screening group was clearly defined by the institutions protocol, with all participants being preoperatively assigned to and undergoing screening as per this strategy, as a result the intervention classification occurred independently of subsequent adverse postoperative outcomes

^4^The study followed a pre-defined institutional clinical pathway, with the results of the intervention leading to expected changes in management such as referral for simultaneous CEA and CABG

^5^The study provided data for the entire 146 patient cohort with all patients being followed up as per the in-hospital postoperative follow up period. There is no evidence of participants being excluded due to missing a screening result

^6^Although assessors were not blinded to screening results, all-cause mortality events are objective events. CVA events although not formally defined did have criteria to be met for formal diagnosis. In this case CVA confirmation via CT imaging was required for formal diagnosis

^7^The study reported findings for the entire 146 patient cohort. There is potential for underreporting of CVA events as a result of the utilisation of the in-hospital postoperative follow up period likely often being shorter than the standard 30-day postoperative period

10. **Hirotani *et al.* 2000**

**Study type:** Prospective cohort study

**Participants:** Patients scheduled for isolated CABG

**Intervention group:** Non-selective screening group (n=472)

**Comparison group:** Selective screening group as per our target RCT

**Outcomes assessed**

- Outcome 1 – Perioperative mortality within the 30-day postoperative period
- Outcome 2 – Perioperative CVA incidence within the 30-day postoperative period

| Outcome | D1: bias due to confounding | D2: bias in selection of participants | D3: bias in classification of intervention | D4: bias due to deviations from intended intervention | D5: bias due to missing outcome data | D6: bias in measurement of outcome | D7: bias in selection of the reported result | Overall risk of bias judgment for outcome |
| --- | --- | --- | --- | --- | --- | --- | --- | --- |
| Outcome 1 | Serious^1^ | Low^2^ | Low^3^ | Low^4^ | Low^5^ | Low^6^ | Moderate^7^ | Serious |
| Outcome 2 | Serious^1^ | Low^2^ | Low^3^ | Low^4^ | Low^5^ | Low^6^ | Moderate^7^ | Serious |

**Reasons for judgements of each domain**

^1^The study lacks a selective screening comparator group, as well as this confounding domains such as aortic calcification and peripheral arterial disease prevalence were not reported. Equally, the reported all-cause mortality and perioperative CVA event counts do not appear to have been statistically adjusted for confounding factors. However, the authors did perform multivariate analyses to identify predictors of stroke

^2^All participants were enrolled for non-selective preoperative CU screening as per the institutions protocol for patients scheduled to undergo non-emergency CABG

^3^The non-selective screening group was clearly defined by the institutions protocol, with all participants being preoperatively assigned to and undergoing screening as per this strategy, as a result the intervention classification occurred independently of subsequent adverse postoperative outcomes

^4^The study followed a pre-defined institutional clinical pathway, with the results of the intervention leading to expected changes in management such as referral for staged CEA and CABG

^5^The study provided data for the entire 472 patient cohort with all patients being followed up as per an in-hospital postoperative follow up period for CVA and a 30-day postoperative follow up period for all-cause mortality. There is no evidence of participants being excluded due to missing a screening result

^6^Assessors were not blinded to screening results, all-cause mortality events are objective events. CVA although not formally defined did have criteria to be met for formal diagnosis. In this case CVA required examination by a neurologist prior to confirmation via CT scanning for formal diagnosis

^7^The study reported findings for the entire 472 patient cohort. There is potential for underreporting of CVA events as a result of the utilisation of the in-hospital postoperative follow up period likely often being shorter than the standard 30-day postoperative period

11. **Bilfinger *et al.* 2000**

**Study type:** Prospective cohort study

**Participants:** Patients scheduled for isolated CABG

**Intervention group:** Non-selective screening group (n=2071)

**Comparison group:** Selective screening group as per our target RCT

**Outcomes assessed**

- Outcome 1 – Perioperative mortality within the 30-day postoperative period
- Outcome 2 – Perioperative CVA incidence within the 30-day postoperative period

| Outcome | D1: bias due to confounding | D2: bias in selection of participants | D3: bias in classification of intervention | D4: bias due to deviations from intended intervention | D5: bias due to missing outcome data | D6: bias in measurement of outcome | D7: bias in selection of the reported result | Overall risk of bias judgment for outcome |
| --- | --- | --- | --- | --- | --- | --- | --- | --- |
| Outcome 1 | Serious^1^ | Low^2^ | Low^3^ | Low^4^ | Low^5^ | Low^6^ | Moderate^7^ | Serious |
| Outcome 2 | Serious^1^ | Low^2^ | Low^3^ | Low^4^ | Low^5^ | Low^6^ | Moderate^7^ | Serious |

**Reasons for judgements of each domain**

^1^The study lacks a selective screening comparator group, as well as this confounding domains such as left main stem disease and intra-operative aortic clamp time were not reported. Equally, the reported all-cause mortality and perioperative CVA event counts do not appear to have been statistically adjusted for confounding factors. However, the authors did perform multivariate analyses to identify predictors of CVA

^2^All participants were enrolled for non-selective preoperative CU screening as per the institutions protocol for patients scheduled to undergo CABG

^3^The non-selective screening group was clearly defined by the institutions protocol, with all participants being preoperatively assigned to and undergoing screening as per this strategy, as a result the intervention classification occurred independently of subsequent adverse postoperative outcomes

^4^The study followed a pre-defined institutional clinical pathway, with the results of the intervention leading to expected changes in management such as referral for simultaneous CEA and CABG

^5^The study provided data for the entire 2071 patient cohort with all patients being followed up as per an in-hospital postoperative follow up period for CVA and a 30-day postoperative follow up period for all-cause mortality. There is no evidence of participants being excluded due to missing a screening result

^6^Although assessors were not blinded to screening results, all-cause mortality events are objective events. CVA, although not formally defined did have criteria to be met for formal diagnosis. In this case CVA required examination by a neurologist prior to confirmation via CT scanning for formal diagnosis

^7^The study reported findings for the entire 2071 patient cohort. There is potential for underreporting of CVA events as a result of the utilisation of the in-hospital postoperative follow up period likely often being shorter than the standard 30-day postoperative period

12. **Gaudino *et al.* 1999**

**Study type:** Prospective cohort study

**Participants:** Patients scheduled for isolated CABG

**Intervention group:** Non-selective screening group (n=2326)

**Comparison group:** Selective screening group as per our target RCT

**Outcomes assessed**

- Outcome 1 – Perioperative mortality within the 30-day postoperative period
- Outcome 2 – Perioperative CVA incidence within the 30-day postoperative period

| Outcome | D1: bias due to confounding | D2: bias in selection of participants | D3: bias in classification of intervention | D4: bias due to deviations from intended intervention | D5: bias due to missing outcome data | D6: bias in measurement of outcome | D7: bias in selection of the reported result | Overall risk of bias judgment for outcome |
| --- | --- | --- | --- | --- | --- | --- | --- | --- |
| Outcome 1 | Serious^1^ | Low^2^ | Low^3^ | Low^4^ | Low^5^ | Low^6^ | Moderate^7^ | Serious |
| Outcome 2 | Serious^1^ | Low^2^ | Low^3^ | Low^4^ | Low^5^ | Low^6^ | Moderate^7^ | Serious |

**Reasons for judgements of each domain**

^1^The study lacks a selective screening comparator group, as well as this confounding domains such as aortic calcification, left main stem disease prevalence and intra-operative aortic clamp time were not reported. Equally, the reported all-cause mortality and perioperative CVA event counts do not appear to have been statistically adjusted for confounding factors. However, the authors did perform analyses to compare outcomes between subgroups of the cohort

^2^All participants were enrolled for non-selective preoperative CU screening as per the institutions protocol for patients scheduled to undergo non-emergency CABG

^3^The non-selective screening group was clearly defined by the institutions protocol, with all participants being preoperatively assigned to and undergoing screening as per this strategy, as a result the intervention classification occurred independently of subsequent adverse postoperative outcomes

^4^The study followed a pre-defined institutional clinical pathway, with the results of the intervention leading to expected changes in management such as referral for simultaneous or staged CEA and CABG, with the decision for patients to receive simultaneous versus staged interventions being based on aortic atherosclerosis levels and whether the patient’s angina was stable or unstable

^5^The study provided data for the entire 2326 patient cohort with all patients being followed up as per an in-hospital postoperative follow up period. There is no evidence of participants being excluded due to missing a screening result

^6^Although assessors were not blinded to screening results, all-cause mortality events are objective events. Intraoperative and postoperative CVA events were given standard definitions. In this case CVA required neurologic examination prior to confirmation via CT scanning for formal diagnosis. It should be noted the cardiac anaesthetist performing the immediate postoperative neurological evaluations was blinded to preoperative screening results

^7^The study reported findings for the entire 2326 patient cohort. There is potential for underreporting of CVA events as a result of the utilisation of the in-hospital postoperative follow up period likely often being shorter than the standard 30-day postoperative period

13. **Birincioğlu C.L. *et al.* 1999**

**Study type:** Prospective cohort study

**Participants:** Patients scheduled for isolated CABG

**Intervention group:** Non-selective screening group (n=722)

**Comparison group:** Selective screening group as per our target RCT

**Outcomes assessed**

- Outcome 1 – Perioperative mortality within the 30-day postoperative period
- Outcome 2 – Perioperative CVA incidence within the 30-day postoperative period

| Outcome | D1: bias due to confounding | D2: bias in selection of participants | D3: bias in classification of intervention | D4: bias due to deviations from intended intervention | D5: bias due to missing outcome data | D6: bias in measurement of outcome | D7: bias in selection of the reported result | Overall risk of bias judgment for outcome |
| --- | --- | --- | --- | --- | --- | --- | --- | --- |
| Outcome 1 | Serious^1^ | Low^2^ | Low^3^ | Low^4^ | Low^5^ | Low^6^ | Low^7^ | Serious |
| Outcome 2 | Serious^1^ | Low^2^ | Low^3^ | Low^4^ | Low^5^ | Low^6^ | Low^7^ | Serious |

**Reasons for judgements of each domain**

^1^The study lacks a selective screening comparator group, as well as this confounding domains such as aortic calcification and intra-operative aortic clamp time were not reported. Equally, the reported all cause mortality and perioperative CVA event counts do not appear to have been statistically adjusted for confounding factors. However, the authors did perform multivariate analyses to identify CVA and severe carotid stenosis predictors

^2^All participants were enrolled for non-selective preoperative CU screening as per the institutions protocol for patients scheduled to undergo CABG

^3^The non-selective screening group was clearly defined by the institutions protocol, with all participants being preoperatively assigned to and undergoing screening as per this strategy, as a result the intervention classification occurred independently of subsequent adverse postoperative outcomes

^4^The study followed a pre-defined institutional clinical pathway, with the results of the intervention leading to expected changes in management such as referral for staged CEA and CABG

^5^The study provided data for the entire 722 patient cohort with all patients being followed up as per the 30-day postoperative follow up period. There is no evidence of participants being excluded due to missing a screening result

^6^Although assessors were not blinded to screening results, all-cause mortality events are objective events. CVA events were given a pre-defined and standard definition. In this case CVA required neurologic examination prior to confirmation via CT scanning for formal diagnosis

^7^The study reported findings for the entire 722 patient cohort

14. **Mickleborough *et al.* 1996**

**Study type:** Prospective cohort study

**Participants:** Patients scheduled for first time isolated CABG

**Intervention group:** Non-selective screening group as per our target RCT

**Comparison group:** Selective screening group (n=1631)

**Outcomes assessed**

- Outcome 1 – Perioperative mortality within the 30-day postoperative period
- Outcome 2 – Perioperative CVA incidence within the 30-day postoperative period

| Outcome | D1: bias due to confounding | D2: bias in selection of participants | D3: bias in classification of intervention | D4: bias due to deviations from intended intervention | D5: bias due to missing outcome data | D6: bias in measurement of outcome | D7: bias in selection of the reported result | Overall risk of bias judgment for outcome |
| --- | --- | --- | --- | --- | --- | --- | --- | --- |
| Outcome 1 | Serious^1^ | Low^2^ | Low^3^ | Low^4^ | Low^5^ | Low^6^ | Moderate^7^ | Serious |
| Outcome 2 | Serious^1^ | Low^2^ | Low^3^ | Low^4^ | Low^5^ | Low^6^ | Moderate^7^ | Serious |

**Reasons for judgements of each domain**

^1^The study lacks a non-selective screening comparator group, as well as this confounding domains such as aortic calcification were not reported. Equally, the reported all-cause mortality and perioperative CVA event counts did not appear to have been statistically adjusted for confounding factors. However, the authors did perform multivariate analyses to identify predictors of CVA

^2^All participants were enrolled for selective preoperative CU screening as per the institutions protocol for patients scheduled to undergo CABG

^3^The selective screening group was clearly defined by the institutions protocol, primarily targeting patients with; prior symptoms of CVA or unexplained neurological symptoms, prior carotid artery disease history, carotid bruit presence or non-palpable carotid pulse. participants were preoperatively assigned to undergo screening as per this strategy, as a result the intervention classification occurred independently of subsequent neurological outcomes

^4^The study followed a pre-defined institutional clinical pathway, with the results of the intervention leading to expected changes in management such as referral for simultaneous CEA and CABG

^5^The study provided data for the entire 1631 patient cohort with all patients being followed up as per a 30-day postoperative period for all-cause mortality and an in-hospital postoperative period for CVA. There is no evidence of participants being excluded due to missing a screening result

^6^Assessors were not blinded to screening results. CVA, although not formally defined did have criteria to be met for formal diagnosis. In this case CVA required diagnosis of CVA via a physician which was then confirmed by CT scanning and a neurologist

^7^The study reported findings for the entire 1631 patient cohort. There is potential for underreporting of CVA events as a result of the utilisation of the in-hospital postoperative follow up period likely often being shorter than the standard 30-day postoperative period

15. **Salasidis *et al.* 1995**

**Study type:** Retrospective cohort study

**Participants:** Patients scheduled for isolated CABG

**Intervention group:** Non-selective screening group (n=387)

**Comparison group:** Selective screening group as per our target RCT

**Outcomes assessed**

- Outcome 1 – Perioperative CVA incidence within the 30-day postoperative period

| Outcome | D1: bias due to confounding | D2: bias in selection of participants | D3: bias in classification of intervention | D4: bias due to deviations from intended intervention | D5: bias due to missing outcome data | D6: bias in measurement of outcome | D7: bias in selection of the reported result | Overall risk of bias judgment for outcome |
| --- | --- | --- | --- | --- | --- | --- | --- | --- |
| Outcome 1 | Serious^1^ | Low^2^ | Low^3^ | Low^4^ | Low^5^ | Low^6^ | Moderate^7^ | Serious |

**Reasons for judgements of each domain**

^1^The study lacks a selective screening comparator group, as well as this confounding domains such as aortic calcification, left main stem disease prevalence and intra-operative aortic clamp time were not reported. Equally, the reported perioperative CVA event count does not appear to have been statistically adjusted for confounding factors. However, the authors did perform univariate and multivariate analyses to identify CVA and severe carotid disease predictors

^2^All participants were enrolled for non-selective preoperative CU screening as per the institutions protocol for patients scheduled to undergo non-emergency CABG

^3^The non-selective screening group was clearly defined by the institutions protocol, with all participants being preoperatively assigned to and undergoing screening as per this strategy, as a result the intervention classification occurred independently of subsequent neurological outcomes

^4^The study followed a pre-defined institutional clinical pathway, with the results of the intervention leading to expected changes in management such as referral for simultaneous CEA and CABG

^5^The study provided data for the entire 387 patient cohort with all patients being followed up as per the in-hospital postoperative follow up period. There is no evidence of participants being excluded due to missing a screening result

^6^Assessors were not blinded to screening results. CVA, although not formally defined did have criteria to be met for formal diagnosis. In this case CVA required confirmation via CT scanning or a neurologist’s assessment for formal diagnosis

^7^The study reported findings for the entire 387 patient cohort. There is potential for underreporting of CVA events as a result of the utilisation of the in-hospital postoperative follow up period likely often being shorter than the standard 30-day postoperative period
