# Supplementary material for: Selective and non-selective carotid ultrasound screening and perioperative stroke incidence in the coronary artery bypass grafting population: a systematic review and meta-analysis
Source: J Cardiothorac Surg. 2026 Jul 19;21:792. doi: 10.1186/s13019-026-04616-6 (PMC13662987; doi:10.1186/s13019-026-04616-6)
Supplement: Supplementary file 4 [file 13019_2026_4616_MOESM4_ESM.docx]

| **Database** | **Combined search string** |
| --- | --- |
| MEDLINE | (("Coronary Artery Bypass"[MeSH] OR "Myocardial Revascularisation"[MeSH] OR "Myocardial Revascularization"[MeSH] OR [CABG[tiab] OR "Coronary Artery Bypass Graft*"[tiab] OR "Myocardial Revascularisation"[tiab] OR "Myocardial Revascularization"[tiab] OR "Coronary Artery Disease"[tiab])) AND (("Ultrasonography, Doppler, Duplex"[MeSH] OR "Carotid Stenosis"[MeSH] OR "Carotid Ultrasound"[tiab] OR "Carotid Duplex"[tiab] OR "Carotid Doppler"[tiab] OR "Carotid Stenosis"[tiab])) AND (("Stroke"[MeSH] OR "Transient Ischaemic Attack"[MeSH] OR "Transient Ischemic Attack*"[MeSH] OR "Cerebrovascular Accident*"[tiab] OR "Transient Ischaemic Attack*"[tiab] OR "Transient Ischemic Attack*"[tiab] OR CVA[tiab] OR TIA[tiab] OR "Perioperative Stroke"[tiab])) |
| SCOPUS | ( INDEXTERMS ( "Coronary Artery Bypass Grafting" ) OR INDEXTERMS ( "Myocardial Revascularisation" OR "Myocardial Revascularization" ) OR TITLE-ABS-KEY ( CABG OR "Coronary Artery Bypass Graft*" OR "Myocardial Revascularisation" OR "Myocardial Revascularization" ) OR TITLE-ABS-KEY ( “Coronary Artery Disease” )) AND ( INDEXTERMS ( "Carotid Duplex Ultrasonography" ) OR INDEXTERMS ( "Carotid Stenosis" ) OR TITLE-ABS-KEY ( "Carotid Ultrasound" OR "Carotid Duplex" OR "Carotid Doppler" OR "Carotid Stenos*" ) ) AND ( INDEXTERMS ( Stroke ) OR INDEXTERMS ( "Transient Ischaemic Attack*" or "Transient Ischemic Attack" ) OR TITLE-ABS-KEY ( "Cerebrovascular Accident*" OR "Transient Ischaemic Attack*" OR "Transient Ischemic Attack*" OR CVA OR TIA OR "Perioperative Stroke" ) ) |
| CENTRAL*² | (CABG OR "Coronary Artery Bypass Graft*" OR "Cardiac Revascularisation" OR "Cardiac Revascularization" OR "Myocardial Revascularisation" OR "Myocardial Revascularization") AND ("Cerebrovascular Accident*" OR "Transient Ischaemic Attack*" OR "Transient Ischemic Attack*" OR CVA OR TIA OR "Perioperative Stroke") |

**Supplementary Table S1.** Search strings utilised across each of the three databases, PubMed, Scopus and CENTRAL.
